# Coseismic fault slip inversion of the 2013 Lushan Ms 7.0 earthquake based on the triangular dislocation model

**DOI:** 10.1038/s41598-022-07458-z

**Published:** 2022-03-03

**Authors:** HuRong Duan, JiaYing Chen, ShuangCheng Zhang, XiaoLong Wu, ZiMing Chu

**Affiliations:** 1 College of Geomatics, Xi’an University of Science and Technology, Xi’an, 710054 Shaanxi China; 2 College of Geological Engineering and Geomatics, Chang’an University, Xi’an, 710064 Shaanxi China

**Keywords:** Geodynamics, Seismology

## Abstract

The 2013 Lushan Ms 7.0 earthquake occurred on the Longmenshan thrust tectonic zone, a typical blind reverse-fault type earthquake that caused the death of nearly 200 people. The investigation of the fault geometry and fault slip distribution of this earthquake is important for understanding the seismogenic tectonic type and seismic activity mechanism of the Longmenshan Fault Zone. In this paper, for the fault geometry of the Ms 7.0 earthquake in Lushan, the geometric parameters of the planar fault are inverted based on the rectangular dislocation model using GPS coseismic displacement data, and on this basis, a curved fault steeply-dipping on top and gently-dipping at depth is constructed by combining the aftershock distribution. The GPS and leveling data are used to invert the slip distribution of the curved fault for the Lushan Ms 7.0 earthquake. The results show that the fault is dominated by reverse slip with a small amount of sinistral rotation, and there is a peak slip zone with a maximum slip of 0.98 m, which corresponds to a depth of ~ 13.50 km, and the energy released is 1.05 × 10^19^ N/m with a moment magnitude of Mw 6.63. Compared with the planar rectangular dislocation model, the curved fault model constructed by using triangular dislocation elements can not only better approximate the fault slip, but also better explain the observed surface displacement, and the root mean square error of the GPS and leveling data fitting is reduced by 1.3 mm and 1.9 mm, respectively. Both the maximum slip and moment magnitude of the fault based on the inversion of the curved structure are slightly larger than the results based on the planar structure.

## Introduction

**Figure 1:**
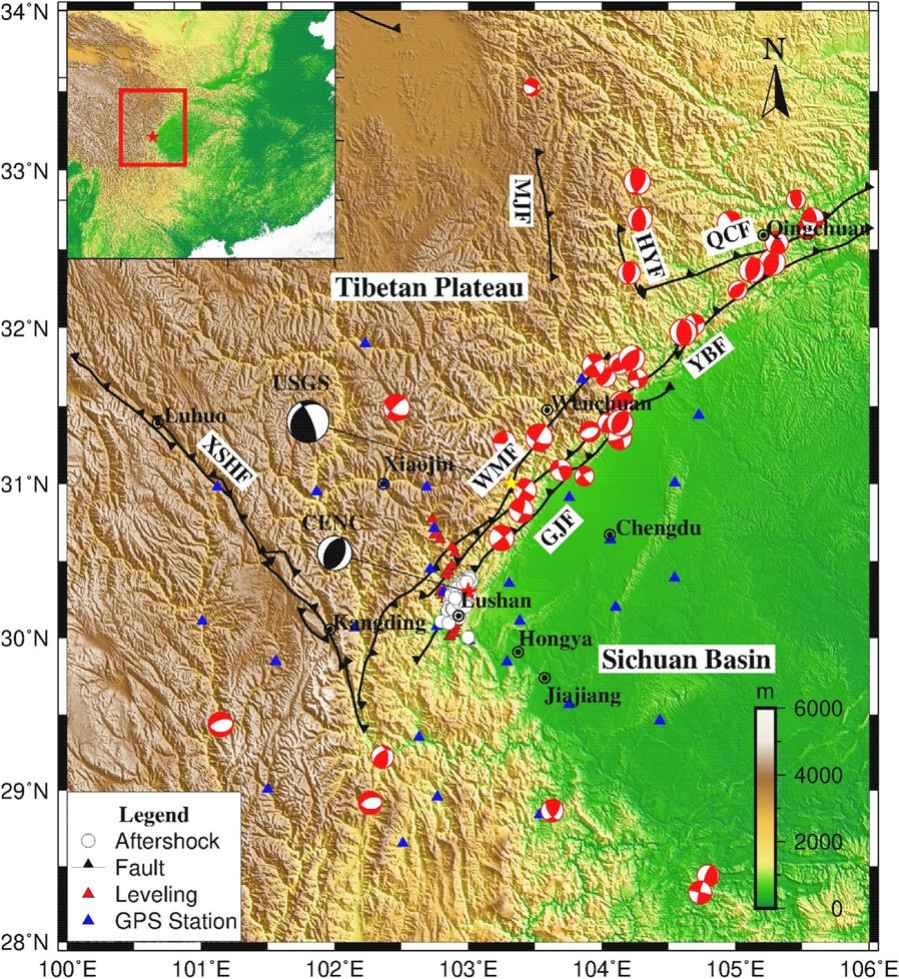
Tectonic background map of Lushan area. The epicenters of the main shocks for the 2008 Wenchuan (yellow star) and 2013 Lushan earthquakes (red star) are shown, along with the aftershocks of the Lushan event (white circles). The blue triangles represent the GPS sites and the red triangles represent the leveling points. Epicenters of earthquakes with Ms > 3.0 between 1976 and November 2021 from the United States Geological Survey (USGS) catalog. Acronyms: WMF: Wenchuan-Maowen fault; YBT: Yingxiu-Beichuan fault; GJT: Guanxian-Jiangyou fault; QCF: Qingchuan fault; MJF: Minjiang fault; HYF: Huya fault; XSHF: Xianshuihe fault. The drawings were prepared using Generic Mapping Tools 5.4.5 (GMT 5.4.5, https://www.generic-mapping-tools.org). The images were overlain on the 30 m Shuttle Radar Topography Mission Digital Elevation Database.

On April 20, 2013, an Ms 7.0 earthquake occurred in Lushan, Sichuan Province, China, causing approximately 200 deaths, injuring more than 12,000 people, and causing significant economic losses within the area affected by the earthquake^1,2^. The epicenter was located at 30.3°N, 103.0°E^3^, which is 80 km south of the epicenter of the 2008 Wenchuan Ms 8.0 earthquake, a thrust earthquake that also occurred in the Longmenshan Fault Zone (Fig. 1). Similar to the Wenchuan earthquake, the Lushan earthquake was a destructive earthquake caused by the southeastward motion of the Tibetan Plateau, which is blocked by the Sichuan Basin, and the compression and collision between these two blocks^4^. However, unlike the Wenchuan Ms 8.0 earthquake, the Lushan earthquake did not form an obvious surface rupture zone and was a blind thrust earthquake according to field observations^5–7^.

Because fault geometry influences earthquake rupture propagation^8,9^, a number of researchers have studied the geometry of the fault and the slip distribution of the Lushan earthquake using seismic waves, leveling data, GPS, and InSAR observations. Previous studies have generally simplified the seismogenic fault to a single inclined plane with a fixed dip angle, and divided the fault into rectangular sub-faults of equal size along the strike and dip to invert the slip distribution^10–17^. They concluded that the earthquake was dominated by a single slip patch, that there was no obvious sign of rupture directivity, that the distribution of coseismic slip was mainly concentrated near the hypocenter, and that there was no obvious slip along the shallow section of the fault, which had an average maximum slip of 1.03 m at a depth of ~ 13.5 km and a moment magnitude (Mw) of 6.6. However, results from aftershock localization, deep seismic reflection profiles, and geomagnetic measurements of the Lushan earthquake^18–23^ indicate that the actual seismogenic fault of the earthquake might have had a complex fault geometry with a steep dipping angle at the top and a low dipping angle at the bottom, and that the oversimplification of the geometry of the seismogenic fault has a significant impact on the reliability of the coseismic slip inversion results, as well as the interpretation of the rupture process and mechanism^24,25^. Some researchers also studied the shovel-like characteristics of the Lushan earthquake, constructing a two-dipping angle fault and flat-ramp-flat models^26,27^ for which the inversion results differed from those of the single inclined surface inversions. Both the two-dipping angle fault and flat-ramp-flat models exhibited double peak rupture zones, in which the maximum rupture was located at 12–15 km and the maximum slip was ~ 1.5 m. Although the abovementioned studies considered the shovel-like characteristics of the rupture surface, they all approximated the shovel-like model by fixing the dip angles of several fault segments, which is a simplified model for shovel-like rupture and does not reflect the continuously changing dip angle with depth^28^.

Some researchers have constructed curved fault models with asymptotic processes along the dip angle, which are more consistent with actual tectonics than a single dipping surface with a fixed dip angle, or the flat-ramp-flat model. Jin et al.^29^ constrained the seismic model using near-field strong motion station observations and GPS data, integrated the spatial distributions of the aftershocks after precise localization, determined the solution of the seismic mechanism and the surface topography, and constructed a curved fault model with a strike of 212° and a linearly graded dip angle that ranged from 54° in the shallow part of the fault to 35° in the deep part of the fault. Xu et al.^30^ used GPS coseismic displacement and leveling data to build a curved fault model for the Lushan earthquake that had a strike of 208° and a dip angle that was asymptotic from 59.4° in the shallow part of the fault to 4.3° in the deep part of the fault, then inverted the fault slip distribution based on the rectangular dislocation model. Duan et al.^31^ used GPS coseismic displacement data to invert the fault dip using the PSO (Particle Swarm Optimization) algorithm and fitted a curved fault model with 3 × 3 fault slices with a strike of 208°, the dip angle variation range is 54°–68° along the strike direction and 37°–68° along the depth direction. The inversion results indicated a maximum slip of 0.82 m at a depth of ~ 13.67 km. The above studies have contributed significantly to understanding the Lushan earthquake, but the rectangular dislocation model was used to perform the coseismic slip distribution inversion. Rectangular dislocations have limitations in modeling the boundaries of complex geometric models, whereas triangular dislocation models are easier to achieve meshing and approximate the geometry of complex faults ^32^. Some researchers have used triangular dislocations to invert the distribution of coseismic fault slip^33–35^. Jiang et al.^36^ found that triangular dislocations are more suitable for the simulation of complex faults than rectangular dislocations in the Yushu earthquake.

To invert for the slip distribution along the Lushan earthquake fault, we used GPS three-dimensional coseismic deformation data to determine the surface deformation field, after which we used the Bayesian method to invert the geometric parameters of the planar fault. By combining the distribution of aftershock data, we use the CFMM (curved fault modeling method) to construct the geometry of the Lushan earthquake curved fault on the basis of this planar fault. In this study, based on the triangular dislocation model combined with a curved fault structure, we inverted the fault slip distribution of the Lushan earthquake using GPS and leveling data. We also discussed the effects of fault geometry (particularly the dip angle), the effect of the shape of the subfaults, and triangular dislocation element size on the inversion results of the slip distribution.

## Data

### GPS data

**Figure 2:**
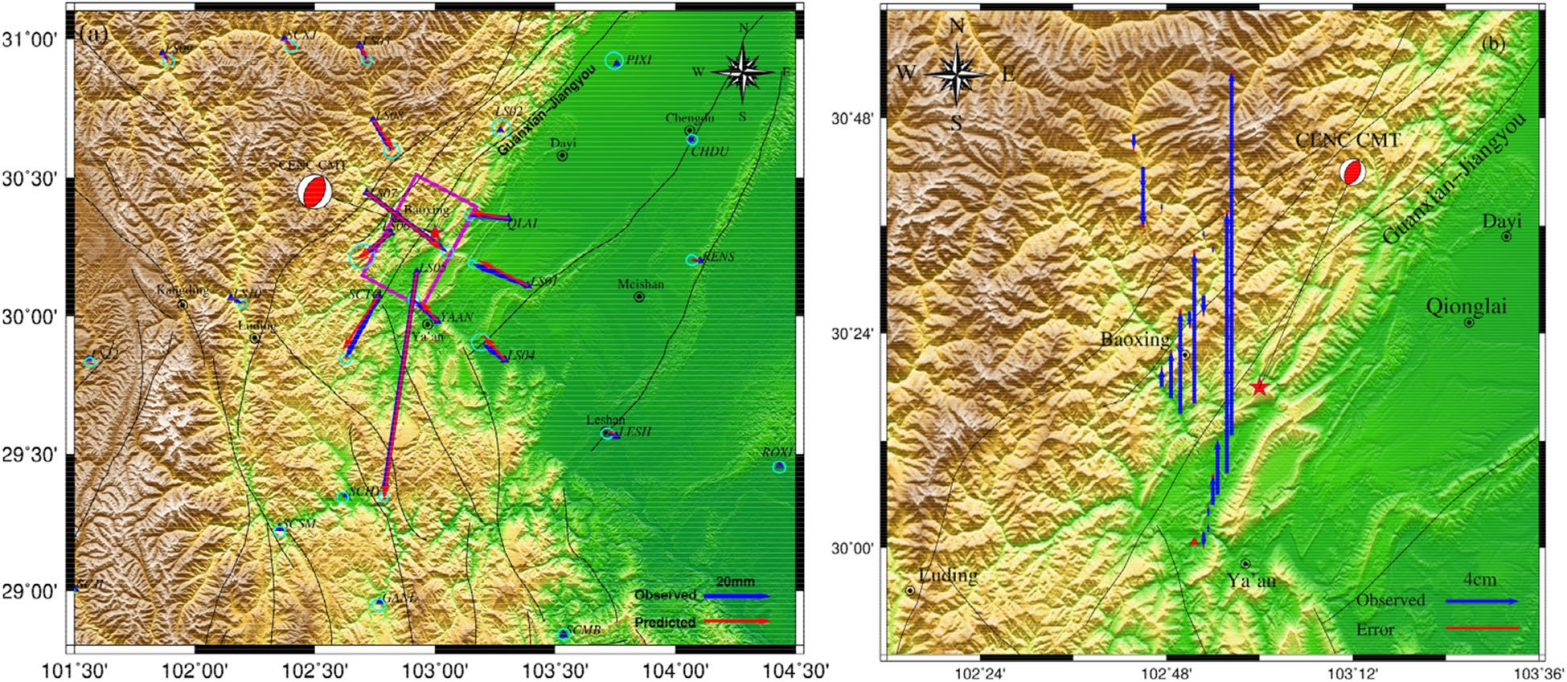
(**a**) The blue vector “Observed” represents the GPS observation, the red vector “Predicted” represents simulated GPS data based on the uniform fault model, the blue triangle represents the GPS continuous station, the pink rectangle represents the ground projection of the Lushan earthquake fault, The red focal mechanisms delineate the fault plane solutions of the Mw 6.6 earthquake, and the blue vector tip ellipse represents the error of the observation data. (**b**) The blue vector “Observed” represents the vertical displacement (relative to MY165 level base point) observed by the leveling station, the red straight line “Error” represents the observation error, and the red triangle represents the MY165 level base point.

After the Lushan earthquake, continuous GPS stations deployed in the surrounding area provided highly accurate data for seismic deformation observations. The coseismic displacement data used in this study were obtained from 33 continuous GPS stations previously described by Jiang et al.^12^ (blue triangles in Fig. 2a, blue vectors represent observed coseismic displacements), and all continuous GPS stations are within 200 km from the epicenter. The deformation variables for points within 150 km from the epicenter were above the millimeter level and were processed using the GAMIT/GLOBK software (http://www-gpsg.mit.edu/~simon/gtgk/). The maximum horizontal error was less than 3 mm and the maximum vertical error was less than 12.4 mm. The maximum displacement point was located at station LS05, which is ~ 15 km south of the epicenter, while the northern and eastern horizontal displacement components were − 66.8 mm and − 9.9 mm, respectively, and the vertical displacement was ~ 83.6 mm.

### Leveling data

The leveling data used in this study were obtained from Hao et al.^37^. The level survey line through the southwestern section of the Longmenshan Fault Zone started at Jiajiang and extended through Hongya, Ya'an, Lushan, and Baoxing to the southeast of Xiaojin, with a total length of ~ 294 km. The sections from Jiajiang to Ya'an and from Ya'an to Xiaojin were observed by the State Bureau of Surveying and Mapping in October 1985 and July 1986, respectively. After the Wenchuan earthquake, the Sichuan Provincial Bureau of Surveying and Mapping used the post-disaster reconstruction project to conduct the first phase of a second-class leveling re-survey along this route in April–October 2010, and re-buried the damaged level points. After the Lushan earthquake, the Second Monitoring Center, China Earthquake Administration conducted a first-class precision leveling survey of the Ya'an–Baoxing survey line through the earthquake area in June–July 2013. The leveling survey line included 22 stations (Fig. 2b), among which station DD35, which is located closest to the epicenter, had a maximum vertical displacement of 198.4 mm.

## Method

### Single planar fault

In geophysical inversion, the inversion results are non-unique because of the uncertainty and finite nature of the observations. Conventional inversion methods have difficulty in fully and correctly characterizing model information in the observed data, and may not adequately represent the inversion results and their reliability^38^. In this study, we used Bayesian software (GBIS; http://comet.nerc.ac.uk/gbis/) to invert the fault geometry. Bayesian inversion provides a natural framework for solving such problems. The inversion algorithm takes into account errors in the data and prior information on model parameters, and aims to rapidly estimate optimal model parameters (Figure S1) and associated uncertainties through efficient sampling of the posterior PDFs (probability density functions). Such sampling is performed using an MCMC ( Markov chain Monte Carlo) method incorporating the Metropolis–Hastings algorithm and with an automatic step-size selection^39^.

We attempted to use a combination of GPS and leveling data to invert the planar fault geometry of the Lushan earthquake. We found that it was difficult to achieve convergence of the fault parameters and the posterior distributions were chaotic (Figure S2), while the fault geometry parameters converged better using the GPS data alone. Therefore, we only used GPS data for the fault geometry inversion. In the inversion process, we restricted the fault dip range from 0° to 90°, the strike range from 180° to 270°, and the number of iterations was set to ${10}^{6}$. The inversion results included nine model parameters: the length and width of the fault, the depth of the lower edge, the dip angle, the strike angle, the coordinates of the midpoint of the sub-fault edge, the amount of slip in the strike-slip direction, and the amount of slip in the dip-slip direction. After several experiments: the inversion results were as follows (the value represent the 2.5% and 97.5% bounds on the posterior probability density functions): the length of the fault was 18.21–24.31 km, the width was 6.48–17.90 km, the dip angle was 41.61°– 44.99°, the depth was 14.64–19.62 km, and the strike was 203.23°–209.57°. Figure 1 shows the GPS observations and surface deformation values orthorectified using the above model. The overall distribution of the deformation field was consistent and the fitting residuals were all within 2 mm. The largest residual was obtained at station SCJL (1.9 mm). Figure 2 shows the posterior probability distribution of the fault geometry parameter inversion using GPS data.

**Figure 3:**
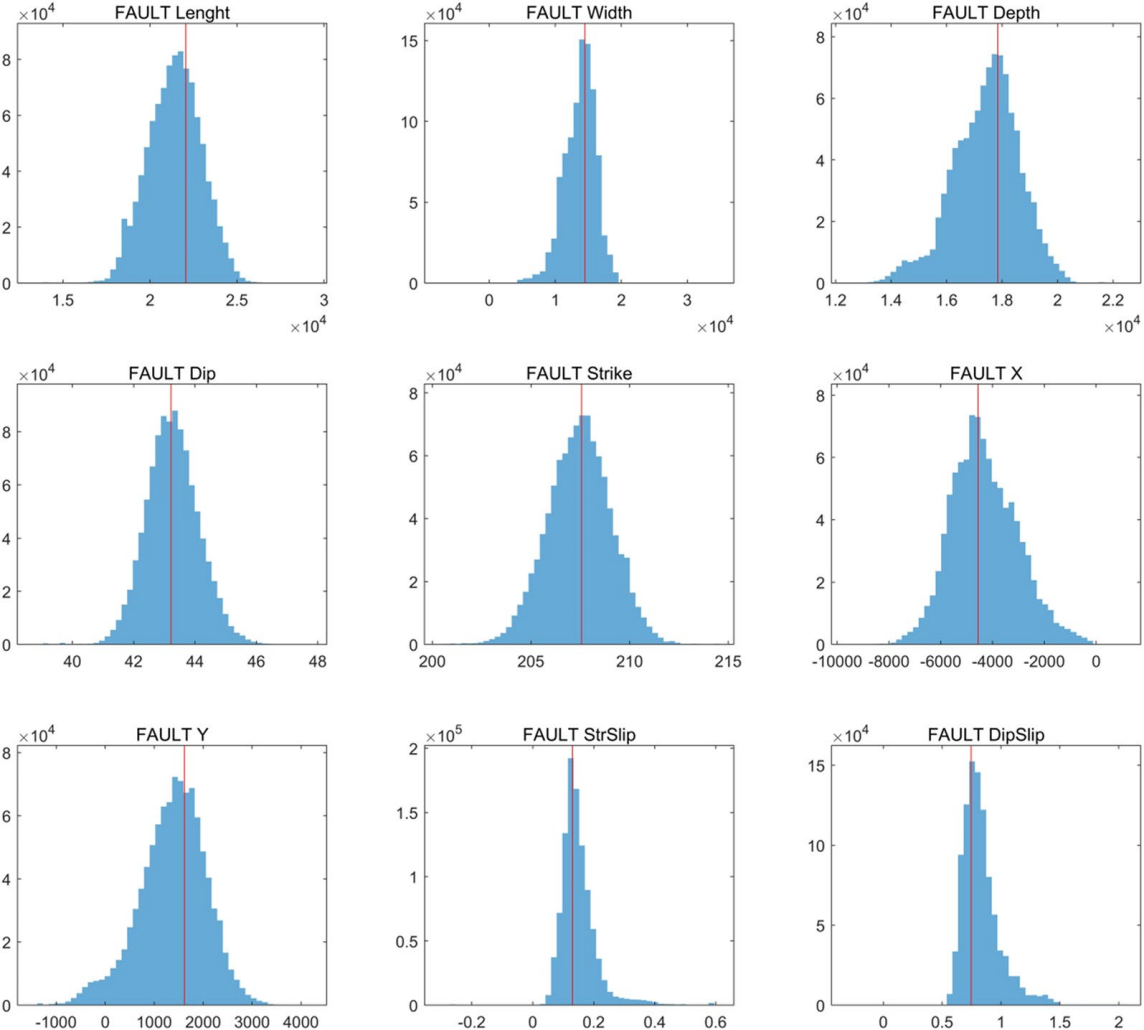
Posterior probability distribution for the fault model parameters. The red line represents the optimal source parameters.

From the geometric parameters of the planar fault presented in Table 1, the best-fit solution were as follows: the length was 22.06 km, the width was 14.60 km, the depth of the lower fault edge was 17.84 km, the depth of the upper fault edge was 7.80 km, the dip was 43.22°, the strike was 207.56°, and the midpoint of the lower edge of the fault (X, Y) was converted to the geographic coordinate system as 30.31° N, 102.89° E. The dip angle obtained from the inversion is consistent with the results of Jiang et al.^12^, which is larger than the dip angles given by CENC (China Earthquake Networks Center) and the USGS. The fault orientation was close to that of Wang et al.^16^. As shown in the posterior probability distribution of the fault geometric parameters (Fig. 3), we obtained solutions for nine fault geometric parameters with approximately normal distribution, which indicates that planar faults can explain the surface data to some extent, but after considering the seismogenic background of the Lushan earthquake, we believe that a single planar fault may not be the optimal choice.

**Table 1:** Geometric parameters of planar faults.

Model parameter	Optimal	Mean	Median	2.5%	97.5%^a^
Length (km)	22.06	21.27	21.43	18.21	24.31
Width (km)	14.60	13.66	13.93	6.48	17.90
Depth (km)	17.84	17.43	17.53	14.64	19.62
Dip( $^\circ$)	43.22	43.26	44.25	41.61	44.99
Strike ($^\circ$)	207.56	207.44	207.49	204.24	210.57
X (m)	− 4560	− 4247	− 4403	− 6620	− 1045
Y (m)	1617	1368	1427	− 230	2600
Fault StrSlip (m)	0.13	0.15	0.14	0.07	0.33
Fault DipSlip (m)	0.75	0.87	0.81	0.62	1.77

^a^We obtained maximum posteriori probability solutions of 2.5% and 97.5% from the posterior probability density functions of the fault parameters.

### Curved fault geometry

Given that the results of the geometric inversion of the planar fault in this paper are very close to those of Jiang et al.^12^, and considering that the rupture may be beyond the scope of the planar fault model, We adopted the method of Jiang et al.^12^ to expand the planar faults. The length of the planar fault is expanded to 46 km, and the width is expanded to 18 km, the dip and strike are set to 43.22° and 207.56° inverted by the Bayesian method. Then, the upper part of the fault is extended to the surface with the dip angle set between 60° and 65°, and the lower part of the fault is extended with the dip angle set between 15° and 20°, and the total width of the upper and lower parts is 20 km^30^. The length of the final three faults constructed along the strike direction is 46 km, and the total width is 38 km, which is consistent with the 46 $\times$ 38 km model finally constructed by Jiang et al.^12^.

**Figure 4:**
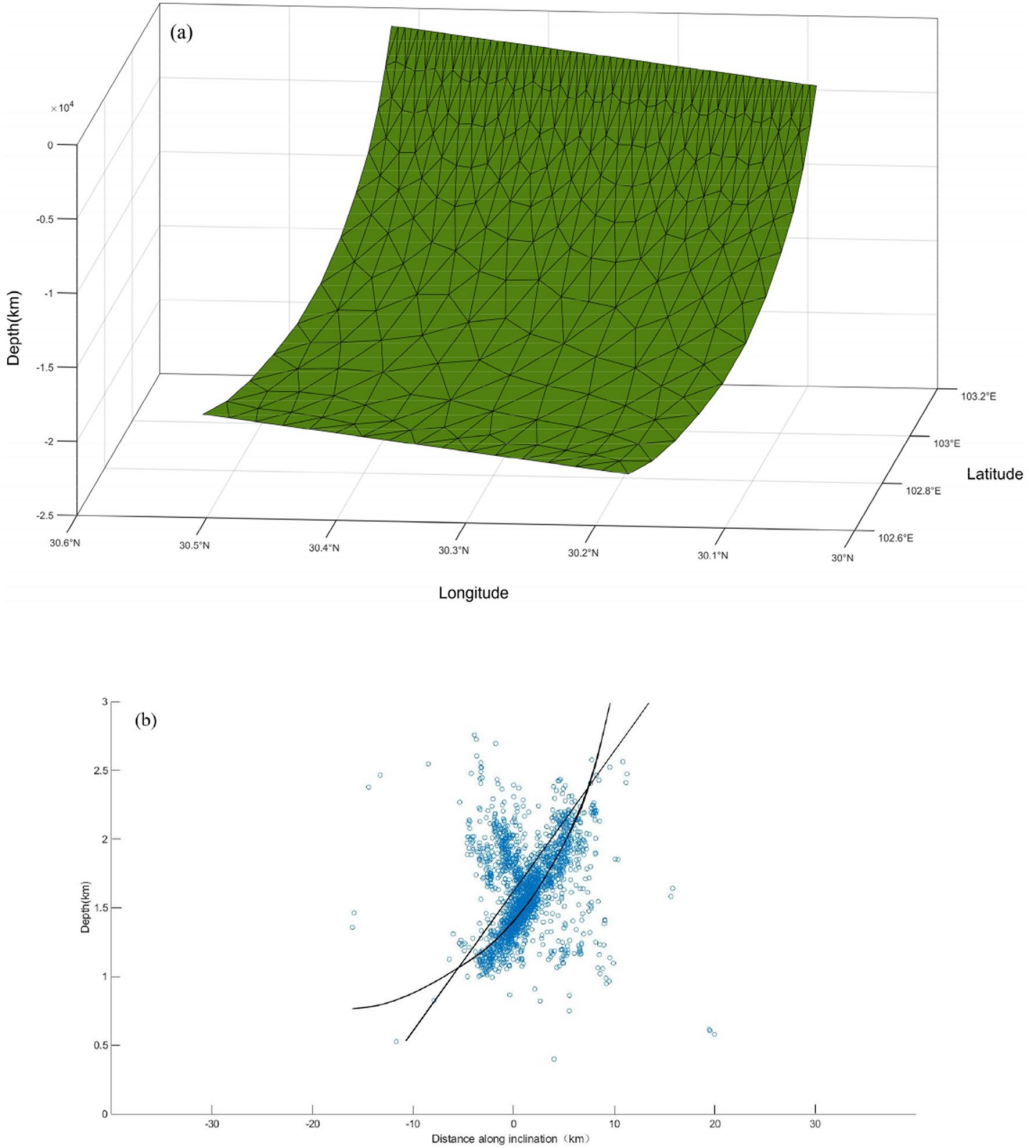
(**a**) Curved fault model of Lushan earthquake; (**b**) The straight line in the figure represents the side view of the tomographic plane, and the curve represents the side view of the curved fault. The blue circle represents aftershocks.

Yin et al.^40^ proposed the CFMM to construct smooth fault surface using triangular elements, with the following goals: (i) construct a 2D irregular triangular network of fault surfaces based on the fault orientation and the locations of the upper and lower boundaries of the fault. (ii) Determine the position and depth of the characteristic inflection point (the dividing point between flat and ramp) on the west and east boundaries of the fault, and use the piecewise cubic Hermite interpolation method to interpolate along the depth direction and strike respectively to construct a smooth quadrilateral auxiliary grid of the fault; (iii) According to the two-dimensional irregular triangulation and the spatial positions of the 4 vertices on each quadrilateral auxiliary grid, the two-dimensional bilinear interpolation method is used to reconstruct the three-dimensional irregular triangulation on each quadrilateral auxiliary grid, and then expand to the entire fault surface to construct a smooth fault triangulation surface^41^. In this paper, the triangular curved fault of the Lushan earthquake is constructed by the CFMM based on the above-mentioned "three faults" structure, and to avoid the sudden change of angle at the junction of the model, which is not consistent with the gradual change of angle of the actual fault along the dip direction. A search interval of 0.1° was used to change the angles of the upper and lower parts of the fault to form a model comprising 2601 faults with different upper and lower angles. The inversion of the slip distribution was conducted based on the principle of a minimum model fitting error. The optimal fault geometry is shown in Fig. 4a, where the fault strike was 207.56°, the fault surface was discretized into 512 triangular dislocation elements, each triangular element had an area of ~ 4 km^2^, the dip of the upper extension was 63.9°, and the dip of the lower extension was 15.8°. In cross-sectional view (Fig. 4b), the majority of the aftershocks occurred in the region near the fault surface and the curved fault structure was located relatively close to the earthquake aftershock sequence^18^, which justifies the curved fault structure.

## Result

**Figure 5:**
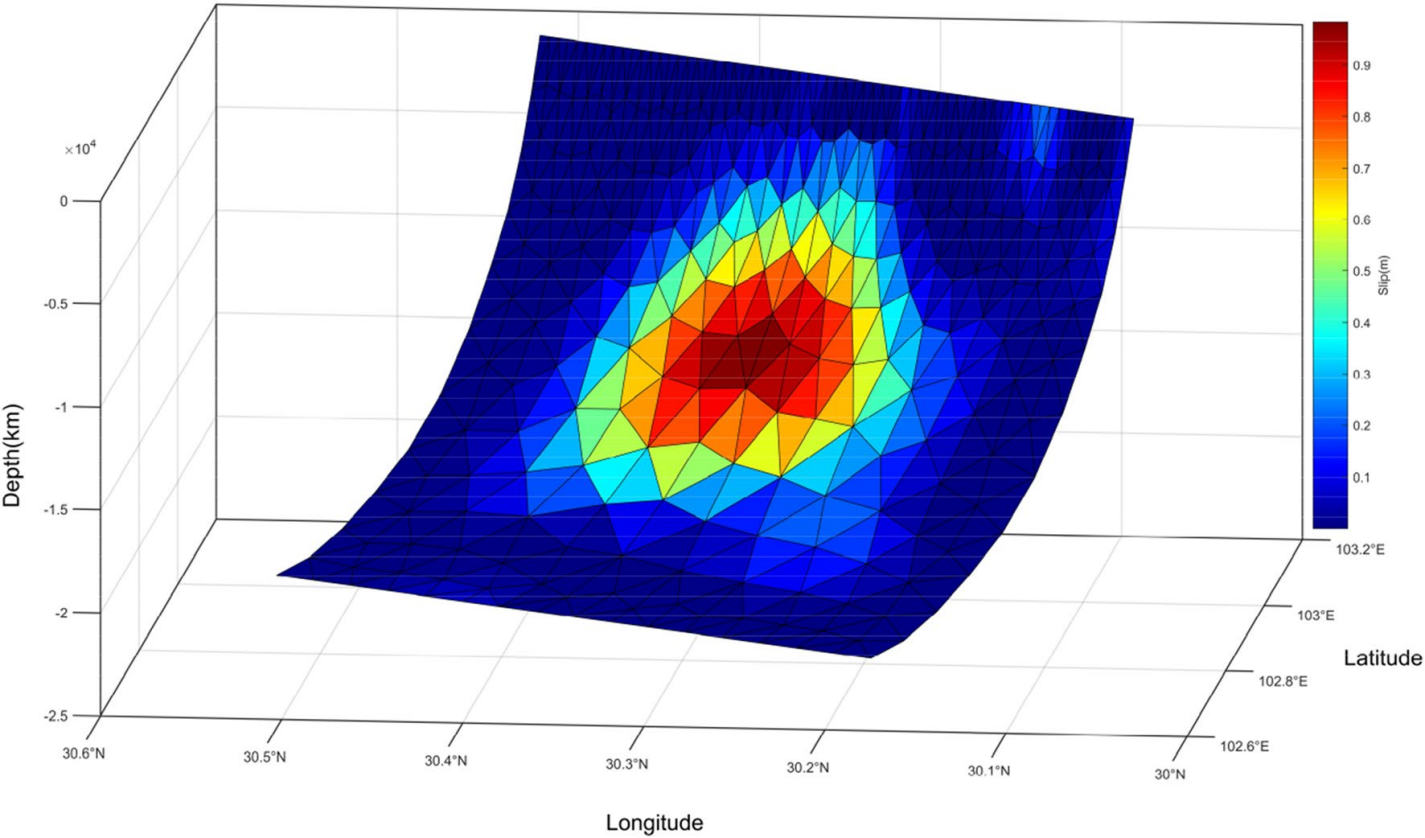
Coseismic slip distribution of the Lushan earthquake.

In this study, a triangular elastic half-space uniform dislocation model was used to construct Green's function between the fault surface slip and surface observations^42^. Then we further inverted for the optimal slip distribution by a variable slip model considering the relative weighting ratio and the smoothing factor (Supplementary III). The inversion results are shown in Fig. 5. The slip distribution of the Lushan earthquake was mainly retrograde, with a small amount of sinistral rotation. The maximum slip was ~ 0.98 m at a depth of ~ 13.50 km, which is larger than the maximum slip obtained by Xu et al.^30^ and smaller than that of the value obtained by Huang et al.^11^ and Chen et al.^26^ (Table 2). The main slip area was located at a depth of 10–17 km, the energy released was $1.05\times {10}^{19}$ N/m (equivalent to Mw 6.63), and the seismic moment and magnitude were the same as those obtained in previous studies. Unlike the double peak rupture zone obtained by Tan et al.^27^, Fig. 5 shows that the rupture of the Lushan earthquake obtained in this study had only one peak rupture zone, which is consistent with the inversion results of Wang et al.^16^ using seismic waves. The main slip was concentrated in a region with a length and width of 20 km, and no large slip occurred within 4 km of the surface.

**Table 2:** Rupture parameters of the Lushan earthquake.

Source	Data	Fault structure	Dislocation element	Maximum slip/m	Magnitude
Jiang et al.^12^	GPS	Plane	Rectangular	0.61	Mw 6.60
Tan et al.^27^	GPS, Strong Motion Data	Double fault	Rectangular	1.50	Mw 6.60
Chen et al.^26^	GPS, Leveling	Flat-ramp-flat	Rectangular	1.20	Mw 6.72
Xu et al.^30^	GPS, Leveling	Curved	Rectangular	0.70	Mw 6.60
Duan et al.^31^	GPS	Curved	Rectangular	0.82	Mw 6.70
Li et al.^13^	GPS	Plane	Rectangular	0.70	Mw 6.60
Mathew et al.^43^	InSAR, Remote sensing data	Double fault	Rectangular	2.26	Mw 6.82
Huang et al.^11^	GPS, InSAR, Leveling, Strong Motion Data	Plane	Rectangular	1.20	Mw 6.53
This paper	GPS, Leveling	Curved	Rectangle	0.98	Mw 6.63

**Figure 6:**
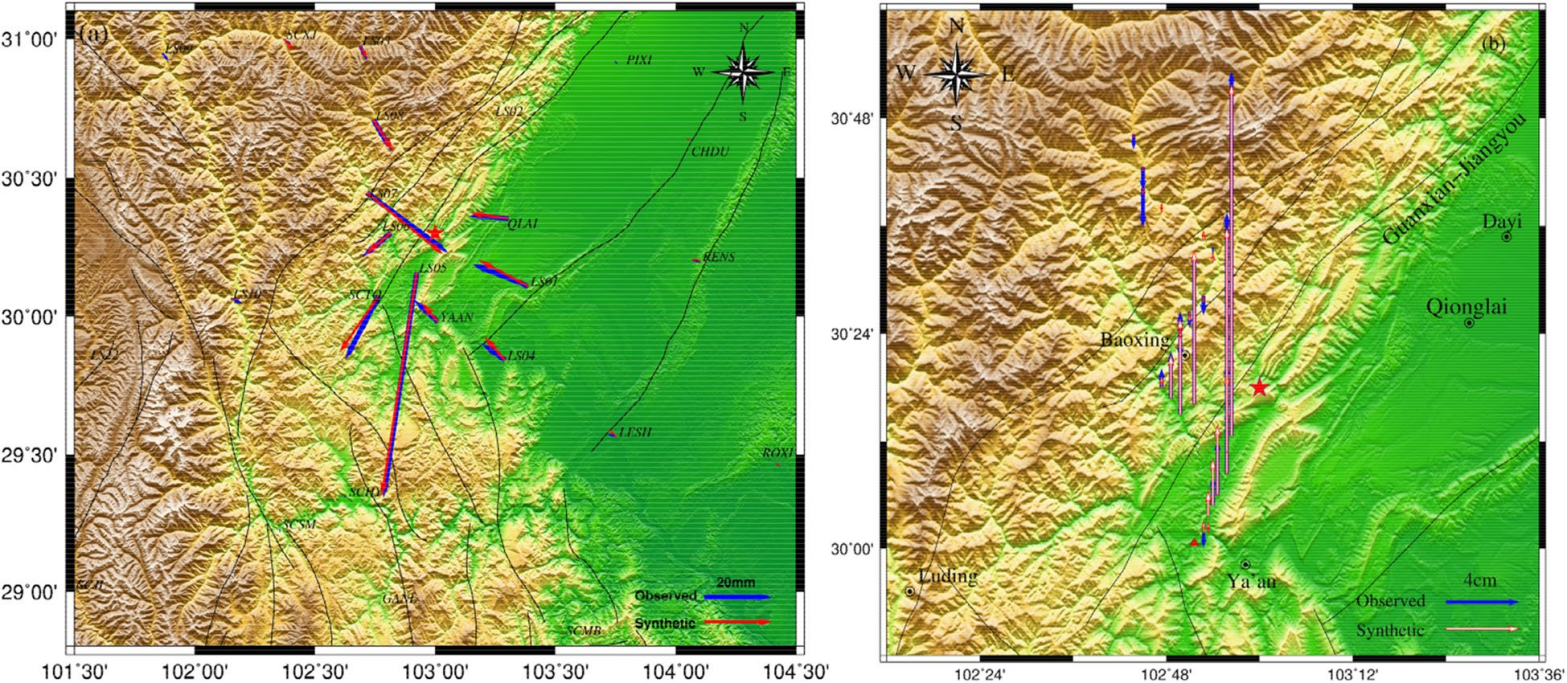
(**a**) The blue vector “Observed” represents the GPS observation, the red vector “Synthetic” represents simulated GPS data based on the finite fault model (triangular curved fault). (**b**) The blue vector “Observed” represents the vertical displacement observed by the leveling station, the red straight line “Synthetic” represents simulated Leveling data based on the finite fault model (triangular curved fault).

From these results, it is clear that the moment magnitudes and maximum slip amounts obtained from the final inversions varied, owing to different data sources and geometric fault parameters used in different studies. To study the fitting results between the fine structure of the fault obtained using a distributed slip inversion and the observed values, we calculated the surface deformation at the observation points using the results of the slip distribution obtained from the inversion (Fig. 6). Figure 6 shows that the predicted values had good consistency for the near-field GPS displacements (e.g., stations LS05, LS06, LS07, etc.), particularly at station LS05, which had the largest horizontal displacement near the epicenter, with a deformation value of 67.5 mm, which is almost the same as the observed value. The residuals at the other stations were generally within 1.5 mm. The root mean square error was 0.28 mm.

The maximum residual for the GPS data was 9.3 mm, which was for the vertical observations at station LS09. The leveling data fitting results are shown in Fig. 6b, for which the root mean square error was 7.6 mm and the maximum vertical displacement was located at station DD35 (198.4 mm, with a residual value of 2.7 mm). The maximum residual was located at station DF17(09) (17.7 mm). The residuals at stations DF16(09) and DF21(09) were also large, likely because the interseismic slip rate of the new station in 2009 was obtained using cubic spline function interpolation which is not the actual observed value^37^.

## Discussion

### Background of the Lushan Earthquake

The vast majority of thrust earthquakes in the world occur on low-angle thrust faults, and the 2008 Wenchuan Ms 8.0 earthquake was the world's first earthquake to appear on a continental high-angle thrust and low slip rate fault. The Tibetan Plateau compresses the west side of the Longmenshan fault zone, and the support of the Sichuan Basin blocks the east side. The 2013 Lushan earthquake and the Wenchuan earthquake have a similar tectonic background. The eastward expansion of the Tibetan Plateau leads to an increase in horizontal compressive stress. If the reverse fault plane is a single high dip angle, the normal stress will increase, and the friction strength of the reverse fault will increase, which is not conducive to the occurrence of slip and strong earthquakes. So how do stress changes on the fault plane cause high-dip thrust faults to slip? If there is a gentle dip angle segment in the deep part of the fault, the friction strength of the entire mild dip angle segment is constant. As long as the stress accumulation reaches this strength, the entire mild dip angle segment will undergo earthquake rupture. The displacement acceleration corresponds to the reduction of the normal stress on the fracture surface. The magnitude of the displacement acceleration is proportional to the reduction of the normal stress on the fracture surface. The slip of the deep fracture leads to a decrease of the normal pressure and an increase of the shear stress on the fracture surface with a high dip angle, which makes the fracture with high frictional strength slip. We believe that this kind of structure with steep top and gentle bottom, the interaction between the deep gentle dip and the shallow steep dip controls the gestation and occurrence of the Lushan earthquake.

### The geometry of the Lushan earthquake fault

Fault geometry is an essential guide for understanding earthquake genesis and mechanisms. The Lushan earthquake was a blind inversion earthquake, and many scholars have a different understanding of the fault geometry of this earthquake. Some results support a planar fault structure with fault dip angles of 43°^12^, 45°^14^, and 42.1°^11^, respectively. Others have proposed segmental fault geometries, such as a flat-ramp-flat geometry with fault surfaces inclined at varying angles of 4°, 35°, and 12°^26^; Tan et al.^27^ constructed a two-dipping angle fault model with an upper dip of 46.8° and a lower dip of 26.5°. Considering the aftershock relocation distribution, Zhang et al.^44^ proposed a "Y" complex structure with four subfaults. In contrast, Qi et al.^24^ suggested the existence of two NW-dipping main faults with a depth range of 8–17 km, two SE-dipping anticline faults, and a possible interface across the Lushan earthquake zone from 8–9 km deep on the NW side to 5–6 km deep on the SE side. The predicted values of these models are highly fitted to the surface observations. Therefore, it is difficult to determine which fault model is optimal under the constraint of the surface coseismic displacement field. After considering the Lushan earthquake's seismogenic background and aftershock distribution, we construct the curved fault as the preferred model based on the upper steep and lower gentle fault structure, whose dip angle is 63.9°–15.8° from the shallow to the deep part.

### Lushan earthquake slip distribution

This paper uses GPS and leveling data to invert the slip model of the Lushan earthquake, revealing the main characteristics. The results are consistent with the previous geodetic inversion results of many scholars. We conclude that thrust faults dominate the rupture of the Lushan earthquake with a large dip angle, and the break of the fault plane has a concentrated slip area near the epicenter, which is consistent with the inversion results of Hao et al.^10^ and Lin et al.^45^ using far-field seismic waves. There is no apparent direction of rupture. The slip distribution solution of the Lushan earthquake shows that this is another thrust event of the Longmenshan fault zone since the 2008 Wenchuan earthquake. The maximum slip occurs at 0.98 m near the hypocenter and at a depth of about 13.5 km, which is close to Duan et al. (0.82 m), but significantly smaller than Zhang et al. (1.61 m)^44^ and Mathew et al. (2.26 m). The total energy released was approximately 1.05 × 10^19^ N/m, which is close to the inversion of Xu et al. (8.77 × 10^18^ N/m), but 37.6% larger than that of Huang et al. (7.63 × 10^18^ N/m) and 41.6% smaller than that of Zhang et al. (1.8 × 10^19^ N/m). There may be two reasons for this discrepancy: the first reason is that the spatial distribution of the observed data, constraints or smoothing conditions may affect the fault slip distribution model; the other reason may be that our model uses the geometry of curved faults for inversion, unlike others, such as Zhang et al. who used four subfaults. In addition, To analyze the effects of fault geometry, subfault shape, and size on the inversion results of the fault slip distribution, we jointly use the GPS and leveling data of the Lushan area to invert the fault slip distribution of this earthquake (Supplementary IV). We compared the residuals of these six models (Table S1). We found that the GPS data fitting residuals were around 3 mm, indicating that the GPS data may not be sensitive to dislocation element types. But for leveling data, our constructed surface faults (B and E) fit better than plane faults. After considering the AIC value and residual error, we believe that the surface fault model constructed by the triangular dislocation element is relatively optimal.

## Conclusions

In this study, the geometric parameters of the planar fault involved in the Ms 7.0 Lushan earthquake were inverted based on the Bayesian method using GPS coseismic displacement data, in which the fault strike was 207.56° and the dip angle was 43.22°. The curved structure of fault involved in the Lushan earthquake was constructed using the CFMM based on a planar fault, with a dip angle that decreased gradually from 63.9° in the shallow part of the fault to 15.8° in the deep part of the fault. The constructed fault surface had a gradual process along the dip angle, which better explained the observed surface deformation than previous fault models.

Based on the curved fault structure, we used the variance component method to invert the coseismic slip distribution of the Lushan earthquake based on the triangular dislocation model by combining the GPS and leveling data. The results indicate that only one peak rupture zone occurred in the 2013 Lushan Ms 7.0 earthquake, for which the rupture directionality was not obvious and the thrust slip was dominated by a small amount of sinistral slip, with the main slip occurring at 10–17 km depths. The maximum slip was 0.98 m at a depth of ~ 13.50 km and the energy released was $1.05\times {10}^{19}$ N/m, which is equivalent to a moment magnitude (Mw) of 6.63.

In this paper, we constructed six different slip distribution inversion models and compared the results. We found that the maximum slip and moment magnitude of the inversion obtained using a simple planar fault model were smaller than those obtained from the inversion using a curved fault structure, which indicates that ignoring the curved fault structure at Lushan might cause the maximum slip and moment magnitude obtained from the inversion to be underestimated under the same data constraints. The fault strike constructed in this paper is single-valued, while it may actually be continuously variable. Our next work will consider this and investigate scientific issues such as coseismic and postseismic stress changes.

## Supplementary Information

Supplementary Information.
