# Supplementary material for: Coseismic fault slip inversion of the 2013 Lushan Ms 7.0 earthquake based on the triangular dislocation model
Source: Sci Rep. 2022 Mar 3;12:3514. doi: 10.1038/s41598-022-07458-z (PMC8894361; doi:10.1038/s41598-022-07458-z)
Supplement: Supplementary file 1 — Supplementary Information. [file 41598_2022_7458_MOESM1_ESM.docx]

### (Ⅰ) Fault geometry parameter

We drew a picture to briefly explain the definition of the 9 parameters (Figure S1). The 9 parameters in the fault geometry are defined as follows: the length (L) and width (W) of the fault, the depth of the lower edge of the fault (Depth), the strike ($\alpha$), the dip (δ), the X and Y coordinates of the midpoint of the lower edge of the fault (shown by the blue dots in the Fig S1), strike-slip in the strike direction (Fault StrSilp), and dip-slip in the dip direction (Fault DipSlip).

**
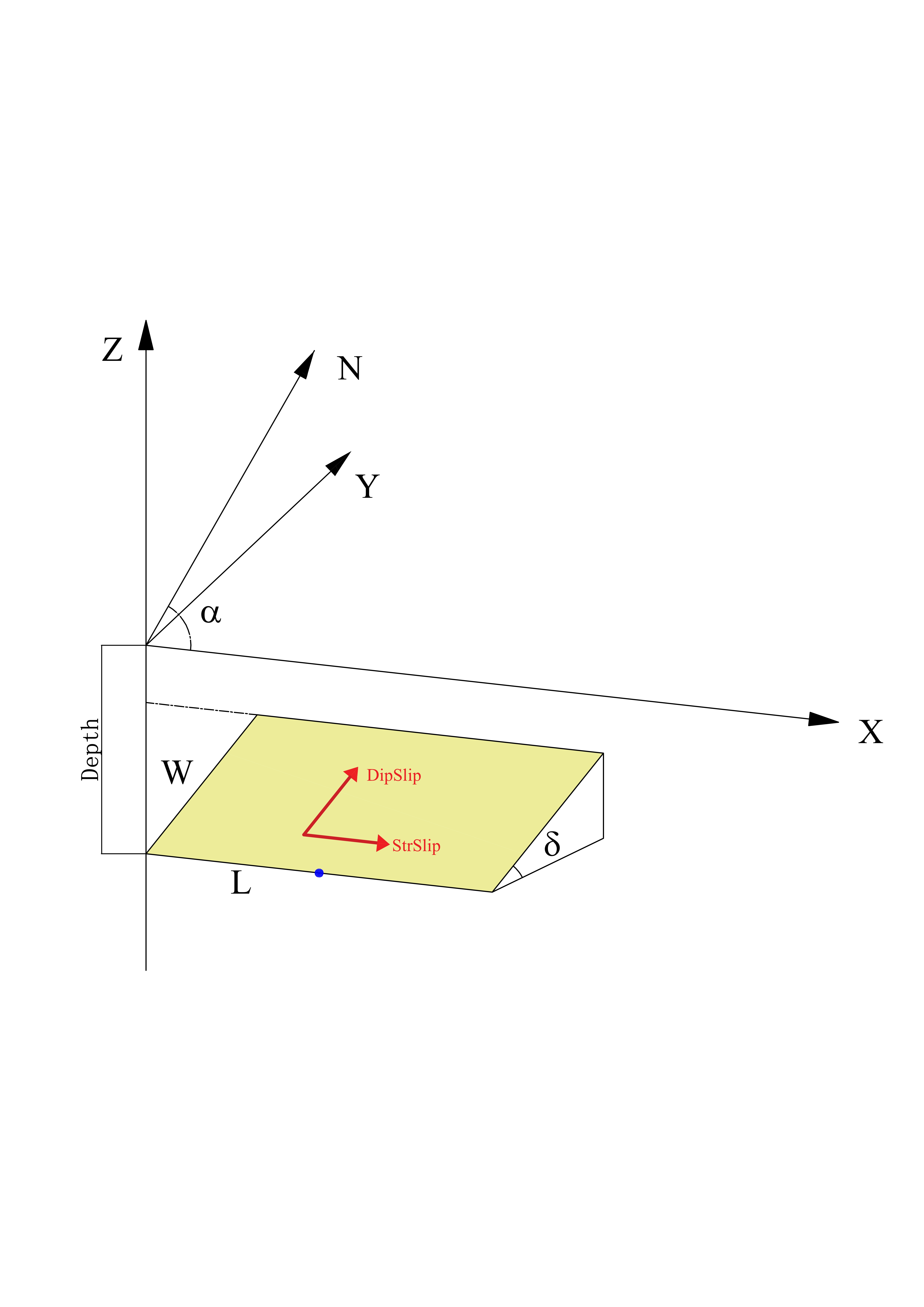
**

Figure S1. Definition of fault geometry in a Bayesian framework

### (Ⅱ) Bayesian inversion results


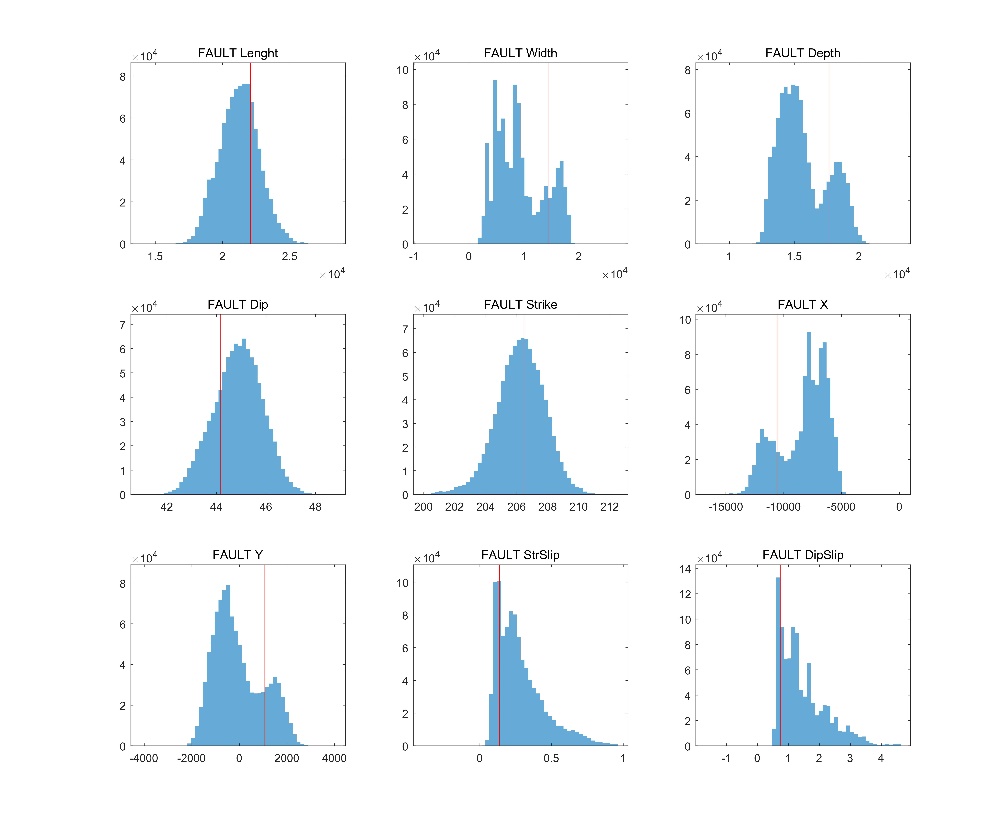


Figure S2. Joint inversion results of GPS and leveling data

### (Ⅲ) Smoothing Factors and Data Weights

To stabilize the inversion results, the Laplace operator was introduced as a smoothing constraint, and the non-negative least squares method was used to solve the relationship between the fault surface slip and surface deformation, the objective function of which is expressed as:

$$\begin{aligned} {\parallel W\left( Gm-d \right)\parallel}^{2}+\beta^{2}{\parallel\left( Lm \right)\parallel}^{2}=min\#\left( S1 \right) \end{aligned}$$

where $G$
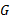
 is the Green's function, $L$
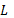
 is the Laplace finite difference operator, $W$
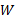
is the weight matrix of the observations, $\beta^{2}$
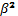
 is the smoothing factor, $d$
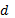
is the surface deformation observations, and $m$
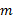
 is the fault slip.


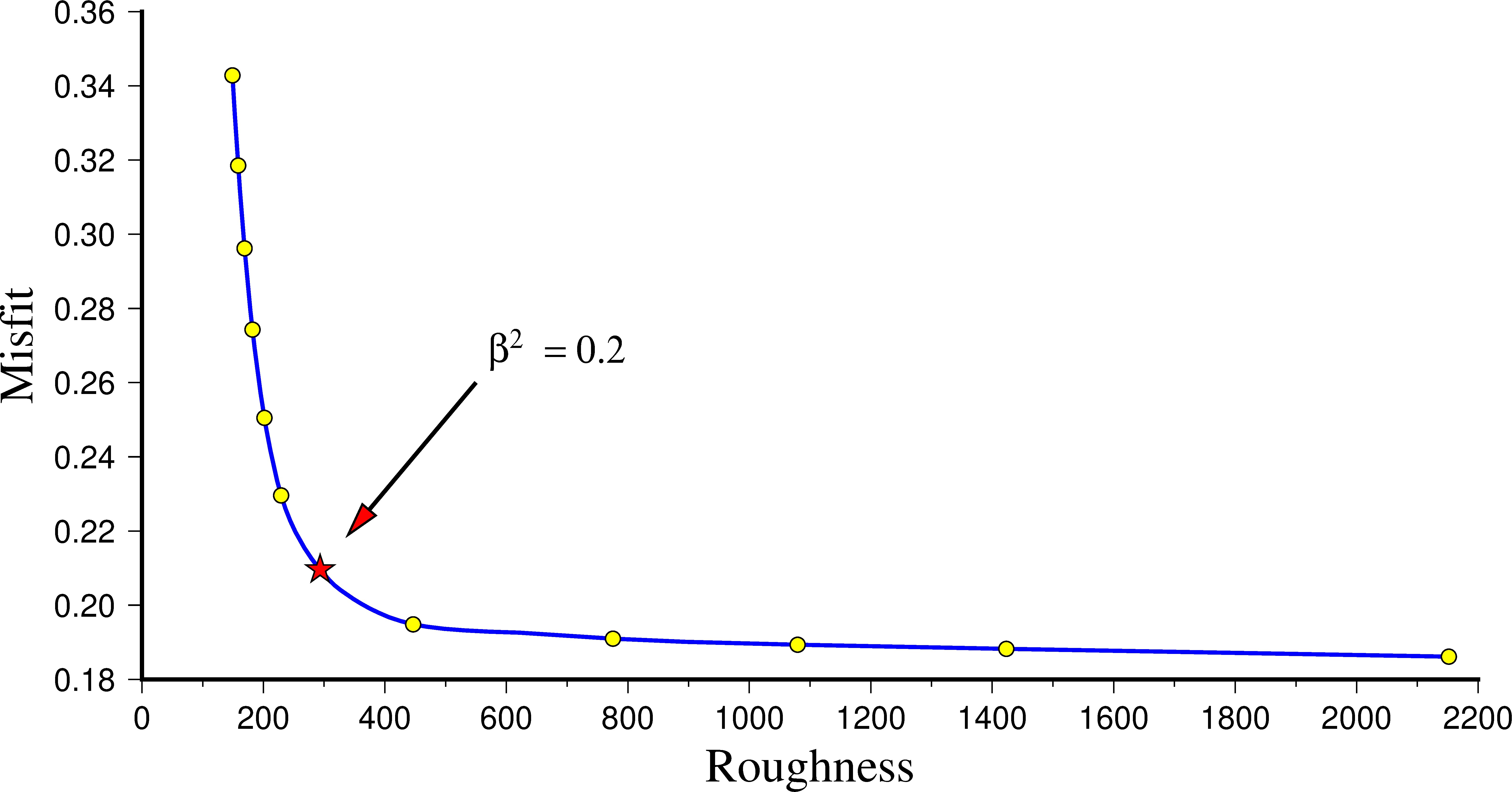


**Figure S3. Trade-off Curve Between Data Fitting Residual and Roughness.**

We used the curved fault structure constructed in Section 3.2 as the geometric structure for the Lushan earthquake fault to invert the slip distribution using the triangular dislocation model. In the joint inversion of the refined fault slip distribution using GPS and leveling data (but we did not consider the error of the data), the relative weight ratio
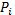
$P_{i}$ between the GPS and leveling data was determined based on the root mean square error
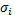
$\sigma_{i}$ corresponding to the data using the Helmert variance component estimation method^S1^. The final weight ratio between the GPS and leveling data was determined to be 1:0.0412. The Laplace smoothing matrix was constructed in the same way as in Barnhart et al. (2010)^S2^. and the smoothing factor
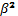
$\beta^{2}$ was determined from the roughness and misfit (fitted residual) curves. By analyzing the relationship between the roughness and misfit, the smoothing factor corresponding to the point near the inflection point (Fig. S3) was set as 0.2.

### (Ⅳ) Slip distribution inversion for different models

we control the number of subfaults at ~550 to ensure the consistency of the number of parameters for each model. The geometries of the faults are discussed when they are planar and curved, and when the shapes of the subfaults are rectangular and triangular, respectively.

### 1 Plane fault

When the Lushan fault is regarded as a plane fault, the dip is 43.22°, the length is 46 km, and the width is 38 km. When dividing faults based on rectangles (Fig. S4 (a)), the size of the sub-faults is ~2×2km, and there are 480 in total. The number of sub-faults using triangular regular division (Fig. S4(b)) and irregular division (Fig. S4(c)) are 540 and 600, respectively. We found that the slip distribution characteristics based on the rectangular dislocation model and the two triangular dislocation models inversion are basically the same. Most of the slip area is concentrated in an area about 20×15 km in length and width, and a rupture peak area is displayed at a depth of ~13 km, which is a typical thrust earthquake (with a small amount of sinistral rotation). The inversion results of the three models have some differences in the slip details. The maximum slip of the rectangular dislocation model, the regular triangular dislocation model, and the irregular triangular dislocation model are ~0.68 m, ~0.97 m, ~0.91m, respectively; The root mean square error of GPS and leveling data fitting are 4.1mm/9.5mm, 3.1 mm/8.8 mm and 3.2 mm/9.0 mm respectively. We found that the maximum slip amount inverted by the triangular dislocation model is obviously larger than that of the rectangular dislocation model, and the root mean square error of GPS and leveling data fitting is smaller. In addition, for plane faults, the triangular division method has little effect on the inverted slip distribution.





**Figure** S4**. Based on the plane fault, the slip distribution inversion of two different dislocation sources. (a) Rectangular Dislocation Source; (b)Triangular Dislocation Source (Regular triangle); (c)Triangular Dislocation Source (Irregular triangle)**

The curved fault has been specifically designed to fit the aftershocks. Therefore, to make a fair comparison with a planar fault, we also design a planar fault that explicitly fits the aftershocks instead of using the Bayesian inversion. We used the Newton-Raphson algorithm to determine the mainshock fault plane parameters by using the spatial distribution of aftershock hypocenter locations^S3^. The fault strike is 208°, the dip angle is 46.3°, and the length and width are set to 46×38 km. The side view is shown in Fig. S5(a) and based on this plane fault, we performed slip distribution inversion (Fig. S5(b)). The maximum slip is ~0.67 m, located at a depth of ~11 km, and the root mean square error of GPS (leveling data) fitting is 9.9 mm (16.8 mm). The above results show that the faults we constructed to adapt to the distribution of aftershocks cannot explain the surface deformation data well.








**Figure** S5**. Plane faults are specially designed for aftershock distribution. (a) Line A represents the side view of the plane fault specially designed for aftershocks; line B represents the side view of the Bayesian inversion fault; curve C is the side view of the curved fault. (b)** **Slip distribution inversion results suitable for aftershock distribution fault.**

### 2 Curved faults

Various data show that the Lushan fault is not a simple planar fault structure^S4-S6^, so it is necessary to construct a curved fault structure to invert the fault slip distribution, and here the dip angle of the curved fault is consistent with Section 3. The fault slip distribution based on rectangular dislocation inversion (Fig. S6), the maximum slip is ~0.74 m, located at a depth of ~13.5 km, and the root mean square error of GPS (leveling data) fitting is 3.3 mm (7.8 mm). Its slip pattern is similar to that of the triangular dislocation model inversion (Fig. 5), and the subfaults slip at both near the surface are less than 0.1 m.





**Figure** S6**. Slip distribution inversion of rectangular dislocation sources based on curved fault geometry.**

To compare the degree of surface displacement explanation by the different fault geometry models, we constructed six different models for slip distribution inversion. Model A is a planar fault with rectangular subfaults. Model B is a curved fault with rectangular subfaults. Model C is a planar fault with regular triangular subfaults. Model D is a planar fault with irregular triangular subfaults. Model E is a curved fault with irregular subfaults. The results are shown in Table S1. We found that the results of all five models show that the main slip of the Lushan fault is concentrated between 10 ~17 km, with only one rupture peak area, and the overall slip characteristics are consistent. From the perspective of fault geometry, the differences between planar and curved faults are shown in models A and B based on rectangular dislocations, with GPS and leveling data fitting residuals of 4.1 mm/9.5 mm and 3.3 mm/7.8 mm, respectively, the latter being significantly better than the former. For models C, D and E based on triangular dislocations, the fitted residuals of GPS and leveling data are 3.1 mm/8.8 mm, 3.2 mm/9.0 mm and 2.8 mm/7.6 mm, respectively, and model E is significantly better than the other two models.

**Table S1 Comparison of different models**

| Model | Dislocation element | Maximum slip/m | GPS RMS/mm | Leveling RMS/mm | AIC |
| --- | --- | --- | --- | --- | --- |
| A(plane) | Rectangle | 0.68 | 4.1 | 9.5 | 2618 |
| B(curved surface) | Rectangle | 0.74 | 3.3 | 7.8 | 2688 |
| C(plane) | Regular Triangle | 0.97 | 3.1 | 8.8 | 2747 |
| D(plane) | Irregular Triangle | 0.91 | 3.2 | 9.0 | 3554 |
| E(curved surface) | Irregular Triangle | 0.98 | 2.8 | 7.6 | 2507 |
| F(plane) ^a^ | Rectangle | 0.67 | 9.9 | 16.8 | 2706 |

^a^ **Planar faults constructed to fit aftershock distribution.**

Considering the different areas of the triangular sub-faults, we calculated the moment distribution diagrams of models A, D, and E (Figure S7). The figure shows that when the rectangular sub-faults is used for division, its area is constant, so the moment distribution map and the slip distribution map are entirely consistent in shape. However, due to the varying size of the triangle, there are some differences between the moment distribution and the slip distribution. Still, in general, the continuity of the moment distribution and the maximum position of the moment release is consistent with the slip distribution.


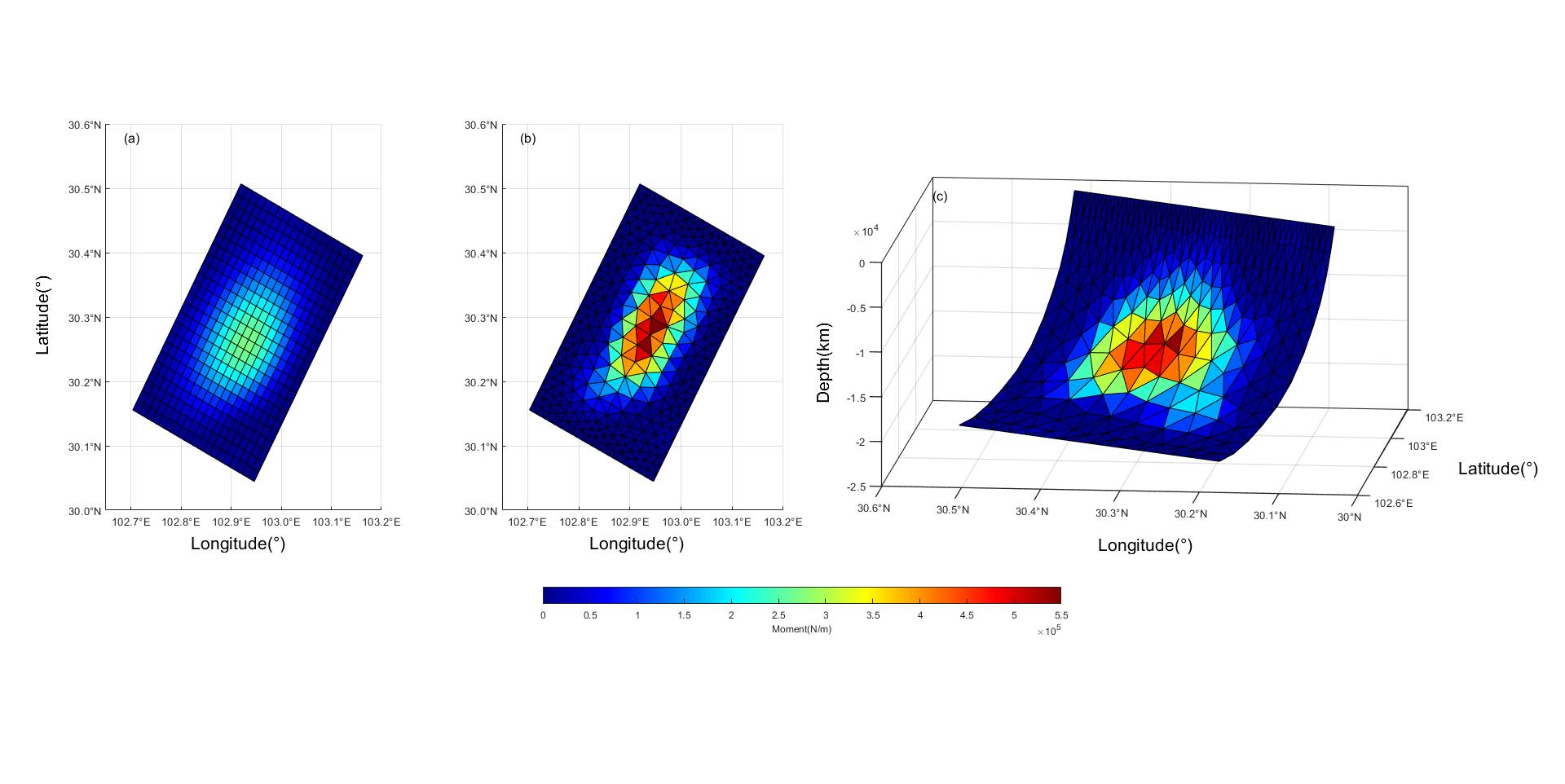
 **Figure** S7**.** **Moment distribution plots for different models. (a) Moment distribution of model A; (b) Moment distribution of model D; (c) Moment distribution of model E.**

### 3 Size sensitivity test for triangular subfaults

To investigate whether the number of subfaults in the surface fault model affects the inversion results, we use the curved faults constructed in Section 3 and divide them into 512, 1114, and 2010 triangular subfaults (Fig. S8), and the maximum slip distributions of the three models are 0.98 m, 1.02 m, and 1.07 m, respectively, and the residuals of the GPS and leveling data fits are 2.8 mm/7.6 mm, 2.8 mm/7.7 mm, and 2.8 mm/7.9 mm, respectively. Figure S8 shows that as the number of subfaults increases, the maximum slip and the residuals of the data fit do not change significantly. This may be due to the excessive subfaults, resulting in insufficient data constraint and pseudo-slip phenomenon. In addition, as the number of sub-faults increases, the number of unknowns also increases. Although the data residuals do not change much, this leads to the AIC value of the surface fault model with 1114 and 2010 subfaults being much larger than that of the surface fault model with 512 subfaults.





**Figure S8. The inversion of the slip distribution of the number of different subfaults based on the triangular curved fault structure. (a) The number of subfaults is 512; (b) The number of subfaults is 1114; (c) The number of subfaults is 2010.**

**References**

s1 Xu, C. J., Ding, K. H., Cai, J. & Grafarend, E. W. Methods of determining weight scaling factors for geodetic-geophysical joint inversion. *Journal of Geodynamics* **47**, 39-46, doi:https://doi.org/10.1016/j.jog.2008.06.005 (2009).

S2 Barnhart, W. & Lohman, R. Automated fault discretization for inversions for coseismic slip distributions. *Journal of Geophysical Research* **115**, doi:https://doi.org/10.1029/2010JB007545 (2010).

S3 Wang, F. C. & Wan，Y.G. The method research using aftershock epicenter distribution to determine the fault plane main earthquake *INLAND EARTHQUAKE*, 39-43, doi:10.16256/j.issn.1001-8956.2007.01.006 (2007).

S4 Burchfiel, B. *et al.* A geological and geophysical context for the Wenchuan earthquake of 12 May 2008, Sichuan, People's Republic of China. *GSA today* **18**, 4-11, doi:https://doi.org/10.1130/GSATG18A.1 (2008).

S5 Jia, D. *et al.* Longmen Shan Fold-Thrust Belt and Its Relation to the Western Sichuan Basin in Central China: New insights from hydrocarbon exploration. *Aapg Bulletin - AAPG BULL* **90**, 1425-1447, doi:https://doi.org/10.1306/03230605076 (2006).

S6 Wang, Z., Huang, R. Q. & Pei, S. Crustal deformation along the Longmen-Shan fault zone and its implications for seismogenesis. *Tectonophysics* **610**, 128-137, doi:https://doi.org/10.1016/j.tecto.2013.11.004 (2014).
